# Support Vector Machine Classifiers Show High Generalizability in Automatic Fall Detection in Older Adults

**DOI:** 10.3390/s21217166

**Published:** 2021-10-28

**Authors:** Jalal Alizadeh, Martin Bogdan, Joseph Classen, Christopher Fricke

**Affiliations:** 1 Department of Neurology, Leipzig University, 04103 Leipzig, Germany; jalal.alizadeh@medizin.uni-leipzig.de (J.A.); joseph.classen@medizin.uni-leipzig.de (J.C.); 2 Department of Neuromorphic Information Processing, Leipzig University, 04009 Leipzig, Germany; bogdan@informatik.uni-leipzig.de

**Keywords:** fall detection, machine learning, SVM, kNN, random forest, older adults, cross-dataset validation

## Abstract

Falls are a major cause of morbidity and mortality in neurological disorders. Technical means of detecting falls are of high interest as they enable rapid notification of caregivers and emergency services. Such approaches must reliably differentiate between normal daily activities and fall events. A promising technique might be based on the classification of movements based on accelerometer signals by machine-learning algorithms, but the generalizability of classifiers trained on laboratory data to real-world datasets is a common issue. Here, three machine-learning algorithms including Support Vector Machine (SVM), k-Nearest Neighbors (kNN), and Random Forest (RF) were trained to detect fall events. We used a dataset containing intentional falls (SisFall) to train the classifier and validated the approach on a different dataset which included real-world accidental fall events of elderly people (FARSEEING). The results suggested that the linear SVM was the most suitable classifier in this cross-dataset validation approach and reliably distinguished a fall event from normal everyday activity at an accuracy of 93% and similarly high sensitivity and specificity. Thus, classifiers based on linear SVM might be useful for automatic fall detection in real-world applications.

## 1. Introduction

Gait impairment and increased risk of falls are frequent complications of numerous neurological diseases like Parkinson’s disease, stroke, and cerebellar disorders [1]. As the prevalence of these disorders typically increases with age, the prevalence of falls increases as well, especially in the aging Western population [2]. Falls are associated with elevated morbidity and mortality, both directly, as a result of the trauma and its consequences, and indirectly, through avoidance behavior, which can lead to inactivity and higher risk of cardio- or cerebrovascular disease [3]. These sequelae may seriously impair the ability to perform daily and social activities, and thus relevantly reduce the quality of life of patients [4,5]. Automatic fall detection allows early notification of caregivers and emergency services and thereby might help in providing rapid medical aid to people who have fallen down.

The availability of high computing power and wearable sensors made automatic fall detection possible. Multiple techniques have been employed previously for this purpose. Fall detection systems (FDS) can be broadly divided into three types: environmental sensing-based systems, wearable sensor-based systems, and vision-based systems [6]. Wearable device-based approaches depend on wearing a small wearable device, usually containing an inertia sensor, placed on the wrist or hip, and are of particular interest due to their unobtrusiveness, wide availability (e.g., in smartphones), low cost, and low power consumption. In order to use these devices for automatic fall detection a number of different algorithms have been proposed (detailed in the following section). In summary, threshold-based algorithms [7,8,9] detect a fall event when a certain parameter, which is computed from the recorded motion data after a specific preprocessing, exceeds a predetermined or rule-based threshold value. As the determination of these cut-off values can be difficult [10], machine learning techniques have been employed which allow purely data-driven classification avoiding the problem arising from the need to predetermine threshold values. It is not sufficient to choose a suitable algorithm that can be trained to detect a fall event in a defined dataset at high accuracy. For later usage in the real world, the generalization to real-world problems is important. However, most algorithms are developed and trained as well as tested on datasets that contain simulated falls and scripted activities of daily living (ADL, [11]). As a first step to generalization, classifiers are frequently cross-validated in different datasets. Because these were also composed of simulated falls [12,13,14], they provide only limited evidence concerning performance in the real world. Only a few studies investigated generalization from training on a fall repository containing simulated falls to testing on real-world accidental falls e.g., by Aziz and colleagues (10 events) [15]. Therefore, testing on real-world fall events appears to be a mandatory step of particular value for the development of reliable FDS [15].

In this study, we investigated whether a number of commonly used machine learning classifiers (RF, SVM, and kNN) show favorable generalizability to real-world data (FARSEEING [16]) when training them on fall repository containing simulated falls (SisFall [17]). We chose these classifiers as they have been shown previously to demonstrate favorable results, and because they are easy to set up and train as well as test given a comparably small number of fall events.

## 2. Related Work

Threshold-based algorithms compute particular parameters from the data stream of the inertia sensors (accelerometers and gyroscopes) and a fall event is detected when a set of parameters under- or surpass some predefined values. To this end, different algorithms have been invented with variable results. Iliev et al. [18] based their calculation on the magnitude of motions recorded and calculated first differences and derivatives on the sum channel of a three-axis accelerometer with reliable results. Quaternions have also been successfully applied in another study by Wu and colleagues [19]. Gyroscope data was used by Zhao and coworkers together with a decision tree algorithm [20]. In order to expand on the amount of information sensor fusion techniques involving magnetometers [21], G-force sensors [22], or circulatory information [23] were used. Threshold-based algorithms have the disadvantage of being dependent on specific parameters and predefined thresholds making them prone to overfitting to the dataset they have been developed on. Indeed, a rather low generalizability of threshold-based approaches was found by Bagala [12] when testing 13 published algorithms on a new fall repository. In order to reduce the impact of manual preselection of parameter thresholds, machine learning was considered a possible remedy. Quadros et al. [24] compared a number of threshold-based algorithms with machine learning approaches and found that the latter generally yielded better results. The major difference yielding this higher potential rests in the capability of machine learning algorithms to detect hidden features automatically in a suitably preprocessed dataset. Setting up the FDS usually involves some kind of training in a supervised or unsupervised learning procedure. The procedure is primarily a classification task and “classical” machine learning approaches (e.g., Support Vector Machine, k-Nearest Neighbor, Random Forest analysis) rely on features calculated by a predefined preprocessing pipeline, which yields a training dataset with known classification results that is used to fine-tune the algorithms. Depending on the choice of the classifier and the sensors used as well as the dataset, different classifiers yielded different results. k-Nearest Neighbor (kNN) and the Least-Squares Method (LSM) worked superior to a Support Vector Machine (SVM) and Artificial Neural Networks (ANN) in a study by Özdemir et al. [25] who processed inputs from an accelerometer and magnetic field data. In Choi et al. [26] a Naïve-Bayes classifier worked best. Another study found the best performance for SVM, kNN, and ANN compared to LSM and Naïve-Bayes [27]. SVM was best in a study by Liu and colleagues [28] while kNN achieved superior results in an event-aligned approach by Putra et al. [29], in detecting falls and falling patterns. Random forest (RF), on the other hand, was the best in a study by Hussain and colleagues [30]. This heterogeneity may result from differences in sensor placement as well as in the dataset itself and its preprocessing. Cross-validation studies involving training classifiers on one dataset while testing them on another may, therefore, be of particular interest to demonstrate the suitability of a classifier in real-world applications. These studies, on the other hand, have been conducted comparably rarely. Igual et al. [13] found that SVM had good generalizability during cross-dataset validation. SVM and ANN were used by Koo et al. [31] in a cross-validation study and showed mixed results. Both studies used fall repositories containing only simulated falls.

Newer but also more involved techniques like Convolutional Neural Networks (CNN) may avoid this feature extraction and uncover hidden structural information solely from the raw data. CNNs, are usually used in image segmentation and classification but have also been used in neurobiological applications in gait analysis [32] as FDS algorithms. Their advantage may lie in avoiding manual feature calculation and selection thus eliminating subjective biases even more so than classical machine learning approaches when compared to threshold-based algorithms. Additionally, they seem to achieve even higher classification accuracy [33,34,35,36]. CNN-derived classifiers have also shown very good generalizability [14] for simulated fall datasets. As a major drawback, these classifiers are comparably difficult to set up, develop, and analyze, they are computationally exceptionally demanding and therefore expensive and the scarcity of fall events even in larger repositories makes training these classifiers more challenging to use for this purpose [35,37]. Apart from these strategies, combinations of threshold-based and machine learning have been used as well in order to increase classification fidelity [38]. In a conceptually different approach, novelty detection with and without personalization using individual motion data was employed by Medrano and colleagues [39,40]. This strategy has the advantage of allowing the development of a suitable classifier in the complete absence of fall events which are then recognized as a novel movement pattern when compared to the known set of ADL which the classifier was developed on. Personalization which involves adding new data to an existing fall repository in order to better train an established classifier has been used by Ngu and coworkers [41] based on the work from Mauldin et al. and this concept may help further improve future FDS classifiers [36]. Ramachandran and Karuppiah [6] give a comprehensive overview of these and other non-wearable-based approaches to FDS systems.

With the FARSEEING database, a large dataset has been published containing more than 100 real-world falls from the elderly and the researchers received 23 datasets on request. A number of FDS algorithms have already been shown to be suitable to detect fall events in this dataset [42,43] though few cross-dataset validations using this real-world fall repository have been published and only a limited number of accidental fall events from other sources were ever incorporated for a cross-dataset validation ([44,45]). In the present study, we used 15 accidental real-world falls from the FARSEEING repository to investigate the generalizability of fall detection algorithms based on classical machine learning techniques which have been trained on a different dataset consisting of simulated fall data (SisFall).

## 3. Materials and Methods

### 3.1. Dataset

**Figure 1:**
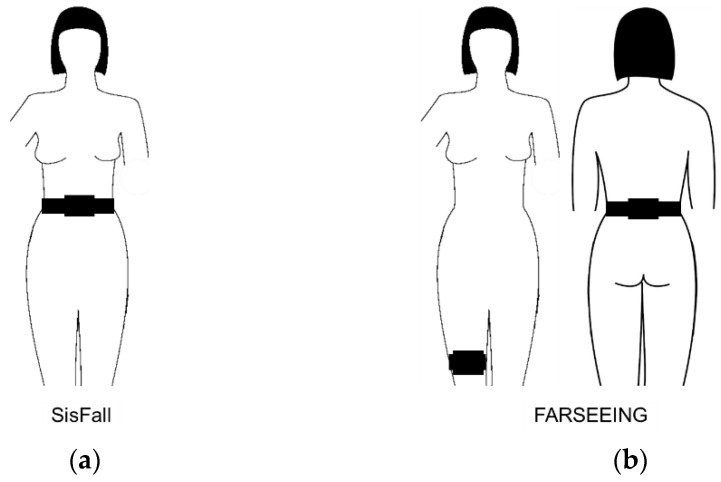
Location of accelerometers during the recording of the two datasets. (**a**) SisFall dataset. Two accelerometers and one gyroscope were attached to a belt around the waist. Only data from one of the accelerometers were analyzed in the present study. (**b**) FARSEEING dataset. One accelerometer was attached to the lower back and the other on the thigh of each participant. Only data from the accelerometer on the lower back was analyzed in the present study. Sensors were selected to achieve the highest similarity with respect to sensor placement.

Two previously published fall repositories have been used for training and validating three machine learning classifiers in this study: SisFall [17] and FARSEEING [16]. Data from the SisFall dataset was used to train and validate the fall detection classifiers. The repository consists of 4510 files containing motion data recorded during 19 activities of daily living (ADL) from 23 healthy young participants (ages 19–30 y.), each of the activities was performed 5 times. The dataset also contains 15 different types of intentional falls which again were conducted 5 times each (Table 1). Additionally, the dataset contains data from 15 older adults (ages 60–75 y.) performing only ADL tasks except one of them also performing intentional falls. In the present study, we used all data from the young participants to train the classifiers, data from the older subject who performed both ADL and intentional falls was used in validating the classification. Data were recorded from three motion sensors (two accelerometers and one gyroscope, all fixed to the waist, Figure 1, left panel) and were recorded at a sampling rate of 200 Hz. We only analyzed the accelerometer to achieve compatibility with the FARSEEING dataset. Table 1 lists the types of falls that have been intentionally performed.

The FARSEEING dataset, which was made available to us upon request, was used to validate the classifiers previously trained on the SisFall dataset but not during training or setting up the classifiers. The available dataset consisted of 23 unique examples of real accidental fall events sampled from 23 older adults (age 56–86 y.). Data were acquired using two accelerometers placed on the lower back and the thigh of the participants, sampled with a frequency of 100 Hz (lower back) and 20 Hz (thigh). For this study, we used only the signals derived by the accelerometer attached to the lower back part of the subjects as shown in Figure 1 (right panel) in order to have a similar sensor position compared to the SisFall data, leaving 15 datasets for the analysis. A previous study has shown that a high performance can be achieved by placing the accelerometer sensors along the longitudinal axis of the body [46] suggesting a suitable sensor positioning although slightly different in both datasets.

We aimed to develop an algorithm useful for the classification of data from a continuous data stream in an online fall detector application. The classifiers were trained on data from the SisFall repository and validated on data from the FARSEEING dataset to demonstrate generalizability.

### 3.2. Data Preprocessing

**Figure 2:**
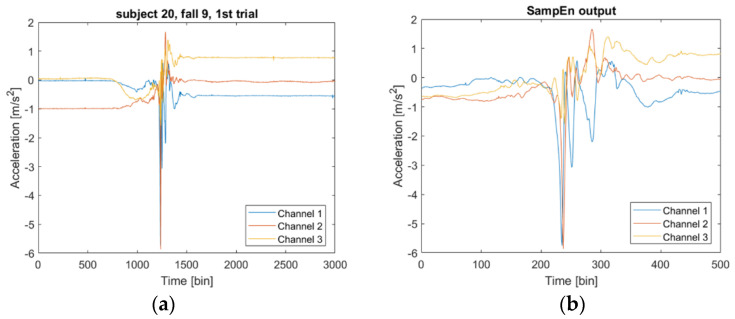
Extraction of a fall event from the SisFall dataset. (**a**) Example of a single dataset containing a fall event (subject 20, fall activity type 9, trial no. 1). The fall event is recognizable between time stamps 1000 and 1500 but there was data unrelated to falls before and after the event of interest. (**b**) With the help of the sample entropy approach, the dataset was cut and reduced to the fall events which was used in further analysis steps. FARSEEING dataset. One accelerometer was attached to the lower back and the other on the thigh of each participant. Only data from the accelerometer on the lower back was analyzed in the present study. Sensors were selected to achieve the highest similarity with respect to sensor placement.

All preprocessing steps were done using Matlab (MathWorks, Natick, MA, USA). We epoched the accelerometer data into non-overlapping segments of 2.5 s length and segments of equal length with 50% overlap. Each trial in the SisFall data had a length of 15 s. For the trials containing intentional fall events, the segments containing the fall event had to be labeled accordingly. This was necessary as a fall event is only of short duration and therefore usually was found in a single 2.5 s segment while the remainder of a trial dataset did not contain immediately fall-related data and therefore was not supposed to be classified as a fall. As there were hundreds of fall trials in the SisFall dataset, we facilitated the identification of the data segments containing falls by using a sample Entropy (SampEn) approach. SampEn is a measure of system complexity [28] and reflects the dynamics of a time series signal. It can be used to detect complex patterns which, in our case, corresponded to the impact associated with a fall event. After epoching, SampEn was computed for each of the segments. Segments were identified as not containing a fall impact and discarded if their SampEn value was lower than a prespecified threshold and kept otherwise, labeled as “fall”. The datasets were thus temporally aligned to the fall impact [31]. The threshold used was empirically determined for all subjects such that it was only surpassed by the SampEn value yielded from a fall impact. We have checked by hand that the detected windows indeed matched with the area of interest (impact) as is illustrated in Figure 2. Note that the SampEn approach is not suitable to classify fall events or non-fall events but is helpful in detecting the precise timing of the fall event in a given limited dataset known to contain such an event. Segments not containing fall events were taken from epoching ADL trials into segments of 2.5 s, labeled as “ADL”. Fall and ADL segments were used to train the classifiers as explained below. In the FARSEEING dataset fall events and ADL were epoched by hand: we labeled 4 s before a fall event as “ADL” and the 2.5 s data segment spanning 1.5 s before and 1.0 s after the impact as “fall”.

We considered the possibility that the performance of the system might depend on the length of the epochs into which the SisFall dataset was divided. Therefore, in addition to a window length of 2.5 s, we investigated the effect of other window lengths on the best classifier.

### 3.3. Classification Algorithms

Three different classifiers were employed to distinguish fall events from ADLs. These were linear Support Vector Machine (SVM [47]), k-Nearest Neighbors (kNN [48]), and Random Forest (RF [49]). SVM is a supervised learning approach extensively used in binary classification tasks and can deal with problems that are either linearly or non-linearly separable. For non-linear problems, SVM computes a hyperplane in a high dimensional space to which the data is virtually transformed. Then, it distinguishes the classes using the hyperplane in the new space and assigns a classification label to a test point based on its location with respect to this hyperplane by assigning the closest input vectors to the dividing hyperplane as so-called support vectors. By contrast, kNN is a non-parametric technique used for both binary and multiclass classification which explores the k closest data points of a training set, with “closeness” abstractly defined as the distance in the relevant feature space. The classification of a test point is achieved by applying the label which is common to the majority of the k-nearest neighbors. The third algorithm applied to the dataset was RF, which is a combination of multiple decision trees allowing us to build a more accurate model than one decision tree alone. It achieves binary and multiclass classification and works very well with categorical features. Each decision tree within RF consists of nodes where each one applies a condition on a feature and decides to which node it should be navigated next to check for another condition. Finally, an output is predicted once a leaf node has been reached where no more conditions can be applied for each of the decision trees in the RF. Therefore, since every decision tree makes its own decision, and the decision can differ, a majority decision is performed at the end of RF, to assign the final class outputted by the RF algorithm. These three classifiers were chosen because they attained a high accuracy related to fall detection in previous studies and support both linear and non-linear solutions [28,29,30,50,51].

### 3.4. Feature Extraction

To train the classifiers and perform the classification, a number of features have been extracted from the datasets. In this study, eight time-domain features and one frequency-domain feature [52] were extracted from each accelerometer channel as well as from the absolute summation (s(t)) of the 3D time series data ($a\left(t\right)=[a_{x}\left(t\right),a_{y}\left(t\right),a_{z}\left(t\right)]$). The features were mean, median, standard deviation, variance, min, max, 0.25 quantile, 0.75 quantile, and spectral entropy. Spectral entropy describes the complexity of acceleration signals and was calculated using Matlab “pentropy” applied after Fast-Fourier Transformation. A total number of 36 features were derived for each subject and type of activity (9 features ×x (3 channels + 1 absolute summation)). In order to have the same sampling rate in all datasets prior to feature extraction, the FARSEEING data was upsampled to 200 Hz using the Matlab function “resample” which employs a FIR antialiasing low-pass filter.

### 3.5. Training of the Classifiers, Classifier Performance, and Statistical Procedures

After preprocessing, the SisFall dataset was divided into a training set (data from 17 out of 23 participants) and a test set (data from the remaining 6 participants). The assignment was random and all trials of a given participant were assigned to either the training or test set. We validated the classifier performance by using the trained classifiers to classify the FARSEEING dataset, corresponding to a transfer learning approach but without the phase of re-training the models, to investigate the generalizability of the algorithms. Additionally, we tested the models on the data from the one elder subject in the SisFall dataset who performed intentional falls to further assess generalizability.

To avoid range effects on the learning algorithm, each feature was standardized to a mean 0 and a standard deviation of 1 prior to the classification. As it might happen that different features show highly similar results, we first applied a feature selection algorithm to reduce the chance of overfitting and increase model performance [34]. Here, we used a sequential backward selection method [53] in order to reduce the number of parameters, computational complexity, and training time. After the feature selection phase, in order to boost the performance of the classifiers, we employed a grid search approach to find optimal hyperparameters for each classifier: for SVM the hyperparameters of C, the kernel scale (for both positive values log-scaled in the range [1E^−3^,1E^3^]) and kernel function; for kNN the constant k (positive integer values log-scaled in the range [1, max (2, round (number of observations/2))]) and the distance metric; for RF the maximum number of features considered for splitting each node (integers in the range [1, max (2, number of features)]), the maximum number of levels (integers log-scaled in the range [1, max (2, number of observations − 1)]), and the minimum number of data points in a leaf node (integers log-scaled in the range [1, max (2, floor (number of observations/2))]) were tuned. Hyperparameters were optimized during training on the SisFall dataset and kept constant for testing on the validation datasets.

We trained the classifiers separately for each of the five trials randomly selected from each of the individuals in the SisFall dataset according to the following workflow: (1) separation of 6 subjects from the SisFall dataset for the SisFall test set, (2) grid search and training of the classifiers using the remaining subjects (SisFall training set), (3) calculation of the classifier’s performance for the SisFall test set and the whole FARSEEING dataset, (4) Repeat steps 1–3, 10 times, and (5) repeat the procedures 1–4 until each of the 5 trials of the SisFall dataset has been used. Each procedure was repeated 10 times in order to evaluate the reliability and variability of the classifier for a number of subset exclusions in step 1. During grid search the following ranges for hyperparameters were selected: for SVM parameter C between 0.01 and 10, for kNN K between 1 and 10, for RF features for node splitting between 1 and 17, the maximum number of levels 30 to 80, and number of data points in leaf node between 1 and 48.

To assess the performance of the classifiers, we calculated the accuracy, specificity, and sensitivity according to the following formulae:

(1) accuracy=(TP+TN)/(TP+TN+FP+FN)

(2) specificity=TN/(TN+FP)

(3) sensitivity=TP/(TP+FN)

where *TP*—true positives (correctly detected fall event); *TN*—true negatives (correctly detected ADL); *FP*—false positives (incorrectly labeled an ADL as fall event); *FN*—false negatives (incorrectly labeled a fall event as an ADL).

Sensitivity reflects the ability to detect a fall event, accuracy the percentage of overall correct classification, while specificity determines the false-alarm rate. Among these, specificity is of particular interest because fall events are much less common than ADL. We employed the non-parametric Kruskal–Wallis test to compare the results among the classifiers. When results were found to be significant we employed Mann–Whitney-U tests as post hoc tests with Bonferroni correction where appropriate. Significance was defined at an alpha threshold of 0.05.

## 4. Results

The results of the classification procedures are presented in Table 2, Table 3, Table 4 and Table 5 where the performance metrics for all SisFall and FARSEEING test sets are shown.

All classifiers achieved high performance metrics when training on the SisFall dataset and testing on a SisFall test set with accuracy, sensitivity, and specificity reaching about 0.9 (Table 2 and Table 3). When comparing individual classifiers in the non-overlapping approach, RF was superior to kNN (*p* = 0.014) and SVM was superior to kNN (*p* = 0.048) in terms of sensitivity, otherwise no significant differences in performance have been found (*p* ≥ 0.05). For the overlapping approach using 50% overlapping epochs of accelerometer data, we found similar results. When testing generalizability to another dataset by using the classifier trained on the SisFall set and using this for classifying the FARSEEING test set, SVM performed significantly better than the other classifiers (both kNN and RF, *p* < 0.01) in all performance metrics while there was a pronounced drop for the other classifiers. This could be observed for the non-overlapping (Table 4) as well as the overlapping approach, which again yielded similar results (Table 5). During each repetition, SVM detected at least 14 out of 15 falls in both approaches. In an additional analysis, we tested the classifiers on the dataset of the single elder subject in the SisFall cohort, who performed both ADL and falls to further assess the generalizability of the SVM. The model produced an accuracy of 0.92 ± 0.01, a sensitivity of 0.82 ± 0.02, and a specificity of 0.99 ± 0.01 (*p* < 0.01 for each of the comparisons), thus yielding comparable results.

**Figure 3:**
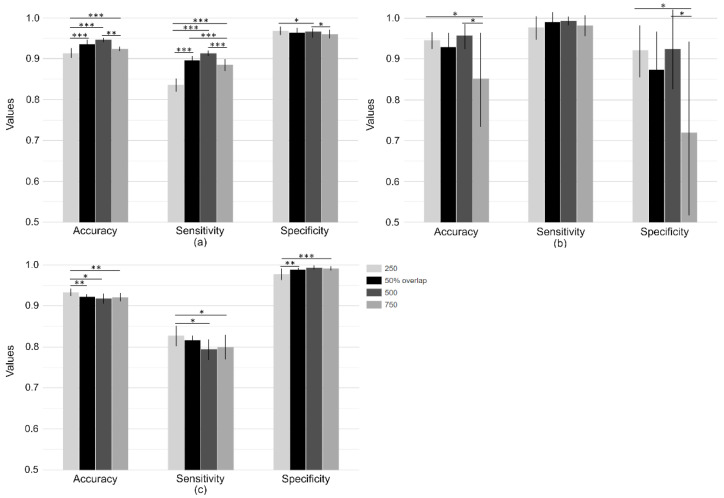
SVM classifier performance metrics across different epoch window lengths. (**a**) Classification of the SisFall test set. (**b**) Classification of the FARSEEING test set. (**c**) Classification of the elder participant in the SisFall dataset performing intentional falls. All window lengths of the non-overlapping approach are compared to one another as well as the 50% overlap approach with window lengths 250 and 750. Light gray, gray, dark gray, and black bars correspond to dataset lengths of 1.25 s, 2.5 s, 3.25 s all with the non-overlapping approach and 50% overlap with lengths of 500, respectively (* *p* < 0.05, ** *p* < 0.01, *** *p* < 0.001).

In order to check whether the length of the time window affected the classifier performance, we tested SVM performance with different non-overlapping window lengths of 250 and 750 data points (equivalent to dataset lengths of 1.25 s and 3.25 s, respectively) and compared it to the performance using segments of a length of 500 data points (overlapping and non-overlapping). According to Figure 3a, SVM for the windows of length 500 data points performed significantly better than for the other two lengths on the SisFall test set in accuracy and sensitivity. With regard to the FARSEEING dataset (Figure 3b), the performance metrics for the windows with a length of 250 or 500 data points (non-overlapping) were significantly superior compared to those obtained with a window length of 750 data points in terms of accuracy and specificity. Considering data from the elder participant in the SisFall dataset (Figure 3c), a window length of 250 was significantly more accurate but showed otherwise a reduced specificity compared to a window length of 500. Thus, we considered a window length of 500 (2.5 s) suitable for the analysis.

In summary, SVM performed best with respect to distinguishing falls from ADL using the same preprocessing steps and feature sets compared to the other classifiers. SVM and RF attained high accuracy, sensitivity, and specificity in distinguishing between ADL and falls in the SisFall test set containing intentional falls only. SVM achieved a significantly higher classification performance than the other classifiers when validated on the FARSEEING dataset containing real-world accidental fall events, indicating the best generalizability.

## 5. Discussion

We investigated three different classifiers (SVM, kNN, and RF) with respect to their utility in automatic fall detection in a cross-dataset validation approach based on two previously published fall repositories containing motion data from intentional (SisFall) and accidental (FARSEEING) fall events. Although all classifiers performed well in distinguishing ADL from intentional fall data taken from the SisFall dataset, SVM worked considerably better than the other tested classifiers (kNN and RF) with respect to generalization to real-world accidental falls as assessed by validating the classifier performance on the FARSEEING dataset. Here, SVM achieved an accuracy of 93 to 96% and a specificity between 87 and 94%.

Our main finding is the high generalizability of the SVM classifier towards a real-world dataset containing accidental fall data. First, the general performance of SVM in our study is consistent with previous work where SVM showed promising results compared to different classifiers [28,54]. Regarding only the classification task in the SisFall dataset, all classifiers performed similarly. This is also in line with previous work demonstrating that—depending on the specifics of the analysis—either SVM, kNN, or RF have yielded the best results [25,26,28]. On the other hand, kNN and RF performed considerably worse in the cross-dataset validation. This could be due to the internal workings of both classifiers, as they tend to overfit to certain features of the dataset used for training resulting in poorer generalization performance. RF is generally considered a very versatile classifier but it features sharp decision boundaries with the tendency to overfit given particular dataset properties [55,56], while kNN is sensitive to data imbalances, characteristics of the distance metrics, and problems in selecting an appropriate value for K [57,58]. Low generalizability is a major problem frequently arising when developing automatic classifiers. To avoid unexpected difficulties when developing for a real-world application, addressing generalization is mandatory. Real-world falls are by default unintentional and may be very unique with respect to their kinematic signature. For example, voluntary movements intended to avoid the fall may vary widely, while the lack of avoidance reactions may make intentional falls more similar to each other. Features extracted from intentional falls may represent the intentional aspect of the falling rather than the falling kinematics. This has led to critique with respect to using intentional falls and might explain why well-working classifiers trained on a laboratory dataset may fail when putting to test in a real-world application [15,59]. This is often reflected in comparably lower performance metrics when during cross-dataset validations or when using real-world data. For threshold-based algorithms, the reduced generalizability has been shown by Bagala and colleagues [12]. For SVM, results have been mixed: Igual and colleagues [13] found that SVM or neural network classifiers worked either well or only moderately depending on the dataset on which they were trained, but no accidental falls were investigated. Similarly, Koo et al. [31] found that SVM and ANN classifiers may work well in internal testing but during external testing only when training data were subjected to a number of preprocessing steps. Of note, they used their own laboratory data for training and validated on the SisFall dataset, but not on accidental falls. Training and testing on real-world data, Palmerini and coworkers [43] used the complete FARSEEING database (more than 100 fall events) and found that a particular SVM setup was superior to other classifiers (kNN, RF, logistic regression, Naïve-Bayes) and a sensitivity of >80% was achieved while still suffering from a false-alarm rate of about 0.5/h. In a cross-dataset approach, SVM was validated by Aziz and colleagues but only on a very small number of accidental falls with a sensitivity of 80% using a more limited set of features [44]. Our study expands and numerically improves on these results by demonstrating the suitability of SVM by validating on a larger dataset. One reason for better performance might have been that training data were impact aligned using the SampEn algorithm. Impact alignment was recently shown to improve classifier performance by Koo and colleagues [31] using a different technique for event detection. Some previously published algorithms reached extremely high performance values up to 100% sensitivity or accuracy (e.g., [60,61]) while others are of similar value as in our study (e.g., [29,42,44,62]). Classifiers and threshold-based algorithms are sometimes subject to overfitting and the dependence of classifier performance on dataset properties has been shown previously [13]. Therefore, very high sensitivities do not necessarily mean that a particular classifier is good overall. For this reason, a cross-validation approach is particularly important. The results for kNN and RF are in line with previous work: A combination of an intentional and real-world dataset to train kNN, RF, Decision Tree, Multilayer Perceptron, and AdaBoost classifiers performed very well when they were trained and tested on the FARSEEING dataset but the accuracy was considerably lower when Silva and coworkers used a dataset with intentional falls for training and the FARSEEING set as a test set [45]. More recently developed machine learning techniques using deep-learning, such as convolutional neural networks, offer feature-less data classification. Indeed, these have been shown to give superior results compared to classical machine learning [36] and have also shown to exhibit good generalizability [14] and thus are of particular interest for future work. As a major disadvantage, these paradigms depend on large datasets for training demanding high computational power which scales with high potencies for improving the classification fidelity [37].

The data preprocessing resulted in a temporally fixed alignment of fall events for the training and testing dataset. There might be the concern that this forces the classifiers to adapt mostly to the timing features of the dataset which might be infeasible in a real-world setting. First, the features we calculated are global values for a whole single trace of data and thus somewhat insensitive to minor changes in temporal alignment. Second, a continuous data stream in a real-life application might use a 2.5 s data buffer which would automatically contain a perfectly aligned temporally falling pattern at some time after (~1.0 s) a fall event when it is continuously updated (first-in-first-out) with newly sampled accelerometer data, triggering the fall detection.

The specificity of our SVM reached 94% for the accidental falls in the FARSEEING repository. Though this number is high and in the order of magnitude previously published in the aforementioned studies, given the relative scarcity of fall events this still means that 6% of ADLs are erroneously classified as fall events even though no such event has happened. Although this fraction of false-alarm rates is similar to the magnitude of 0.1 to 0.5/h [31,43], this limits the immediate utility of the classifier. A possibly superior classification fidelity might have been achieved using a larger number of datasets during training using additional fall repositories [11] although SVM has been known to work well on small datasets. Another limitation of our study is the small size of the real-world fall dataset used for cross-validation. We tried to address this by including the intentional fall data from the elder subject in the SisFall dataset which was otherwise not regarded during the training procedure which yielded similar results when evaluating the SVM model, but further work should include larger real-world datasets for validation purposes.

## 6. Conclusions

We found that a SVM classifier yielded the best performance metrics when trained on a fall dataset containing intentional falls and cross-validating it with real-world falls. Results were robust, and accuracy, sensitivity, and specificity were high enough to make the classifier appear suitable for further investigation with respect to real-world applications. A future aim will be to not only detect but also prevent falls. This will likely require testing the generalizability of the present classifier on additional datasets and solving prediction problems when trying to achieve reliable pre-impact fall detection.

## Figures and Tables

**Table 1:** Types of intentional falls contained in the SisFall dataset.

Activities	Activities
Fall forward while walking caused by a slip	Fall backward while walking caused by a slip
Lateral fall while walking caused by a slip	Fall forward while walking caused by a trip
Fall forward while jogging caused by a trip	Vertical fall while walking caused by fainting
Fall backward while sitting, caused by fainting or falling asleep	Fall forward when trying to get up
Lateral fall when trying to get up	Fall forward when trying to sit down
Fall backward when trying to sit down	Lateral fall when trying to sit down
Fall forward while sitting, caused by fainting or falling asleep	Lateral fall while sitting, caused by fainting or falling asleep
Fall while walking, with use of hands on a table to dampen fall, caused by fainting	

**Table 2:** Performance metrics for each classifier, SisFall test set for non-overlapping approach (0% overlapping epochs). All classifiers have been trained on a SisFall set and evaluated using a SisFall test set, from which the metrics have been calculated. Each classifier yielded similarly high classification metrics. The values are mean ± standard deviation.

Variable	SVM	kNN	RF
Accuracy	0.93 ± 0.01	0.92 ± 0.01	0.94 ± 0.01
Sensitivity	0.89 ± 0.02	0.87 ± 0.02	0.91 ± 0.01
Specificity	0.96 ± 0.01	0.97 ± 0.01	0.97 ± 0.01

**Table 3:** Performance metrics for each classifier, SisFall test set for overlapping approach (50% overlapping epochs). All classifiers have been trained on a SisFall set and evaluated using a SisFall test set, from which the metrics have been calculated. Each classifier yielded similarly high classification metrics. The values are mean ± standard deviation.

Variable	SVM	kNN	RF
Accuracy	0.93 ± 0.01	0.94 ± 0.01	0.94 ± 0.01
Sensitivity	0.90 ± 0.01	0.91 ± 0.01	0.90 ± 0.01
Specificity	0.97 ± 0.01	0.96 ± 0.01	0.97 ± 0.01

**Table 4:** Performance metrics for each classifier, FARSEEING test set for non-overlapping approach (0% overlapping epochs). All classifiers have been trained on a SisFall set and evaluated using a FARSEEING test set, from which the metrics have been calculated. SVM achieved the best results with the highest classification metrics. The values are mean ± standard deviation.

Variable	SVM	kNN	RF
Accuracy	0.96 ± 0.02	0.75 ± 0.10	0.60 ± 0.03
Sensitivity	0.98 ± 0.04	0.76 ± 0.11	0.92 ± 0.07
Specificity	0.94 ± 0.04	0.74 ± 0.20	0.27 ± 0.04

**Table 5:** Performance metrics for each classifier, FARSEEING test set for overlapping approach (50% overlapping epochs). All classifiers have been trained on a SisFall set and evaluated using a FARSEEING test set, from which the metrics have been calculated. SVM achieved the best results with the highest classification metrics. The values are mean ± standard deviation.

Variable	SVM	kNN	RF
Accuracy	0.93 ± 0.05	0.75 ± 0.07	0.58 ± 0.02
Sensitivity	0.99 ± 0.03	0.78 ± 0.12	0.95 ± 0.07
Specificity	0.87 ± 0.10	0.73 ± 0.10	0.22 ± 0.06

## Data Availability

The fall repositories used in this study can be acquired online at http://farseeingresearch.eu/ (FARSEEING) and http://sistemic.udea.edu.co/en/investigacion/proyectos/english-falls/ (SisFall).
